# 科研成果转化为教学资源对提升教学效果的作用:以药物分析学课程中色谱方法的教学为例

**DOI:** 10.3724/SP.J.1123.2023.12020

**Published:** 2024-05-08

**Authors:** Ligai BAI, Bin LIU, Xiaoqiang QIAO

**Affiliations:** 1.河北大学药学院, 药物化学与分子诊断教育部重点实验室, 河北 保定 071002; 1. Key Laboratory of Medicinal Chemistry and Molecular Diagnosis of Ministry of Education, College of Pharmaceutical Sciences, Hebei University, Baoding 071002, China; 2.河北大学党委研究生工作部/研究生院, 河北 保定 071002; 2. Party Committee Graduate Work Department/Graduate School, Hebei University, Baoding 071002, China

**Keywords:** 科研成果转化, 教学资源, 色谱方法, 药物分析学课程, 教学效果, 问题驱动, transformation of scientific research achievements, teaching resources, chromatographic method, pharmaceutical analysis course, teaching effect, problem-based learning

## Abstract

药物分析学课程是将多个课程联系起来形成新的综合知识节点的三维知识网,涉及知识体系庞大,知识结构灵活。其中,色谱分析法在这门课程中涉及面极广,要准确掌握其内容难度很大,这会导致教学效果不好。本着立德树人的教育宗旨和培养复合型高素质专业人才的目标,本文以药物分析学课程中色谱方法的教学为例,将与色谱方法相关的科研成果转化为教学资源,融入教学过程,将创新理念和科研思路渗透给学生,这不仅有利于学生理解并掌握知识,而且能够锻炼学生提出问题和解决问题的能力。本文进一步阐述了如何通过课程设计引入科学发展前沿并渗透科学问题,从而阐明将科研成果转化为教学资源对达成教学目标的促进作用。针对药物分析学课程知识更新速度快、理论与实践并重的特点,我们通过引入色谱科学发展前沿、渗透科学问题、采用问题式与任务驱动式相结合的教学方法对课程进行设计,并采用多元化考核、学生反馈、自我评价3个方面对教学效果进行评估。结果表明,将科研成果转化为教学资源对激发学生学习兴趣、提升学生解决问题的能力,以及达成课程目标具有显著作用,在很大程度上提升了教学效果,实现了学生知识、能力和素质的共同提高。

## Abstract (en)

The pharmaceutical analysis course is a three-dimensional knowledge network that connects several courses to form a new comprehensive knowledge node involving a large knowledge system and flexible knowledge structure. In this course, the subject of chromatography covers a wide range of topics. However, because accurate content is challenging to present, the teaching effect of this subject is poor. In this work, we sought to achieve the educational purpose of establishing morality and cultivating talent, as well as the goal of training highly skilled professionals, by taking the teaching of chromatography in the pharmaceutical analysis course as an example of transforming scientific research results into teaching resources. The resources obtained are integrated into the teaching process to provide innovative and scientific research ideas to students with the aim of not only helping them understand and master technical knowledge but also exercise their ability to raise and solve problems. Furthermore, we expound on how to introduce scientific development frontiers and formulate scientific problems through curriculum design. We also describe how our strategy can promote the teaching effect and achieve teaching objectives. Based on the characteristics of rapid knowledge update and equal emphasis on theory and practice in pharmaceutical analysis, the course is designed by introducing new advances in scientific development, formulating scientific problems, and adopting question- and problem-based learning methods for teaching. The teaching effect is then evaluated through diversified assessment, student feedback, and self-evaluation. The results show that the transformation of scientific research results into teaching resources plays a significant role in stimulating students’ interest in learning, improving students’ ability to solve problems, and achieving curriculum objectives, all of which greatly improve the teaching effect.

尽管我国高等教育的国际影响力不断扩大,但是大而不强、活力不够、重科研轻教学等问题依然比较突出。人才是衡量一个国家综合国力的重要指标,也是创新活动中最为活跃、最为积极的因素^[1]^。没有人才优势,就不可能有创新优势、科技优势、产业优势,因此培养创新人才是国家科技发展的驱动力。与此同时,为了提升高校的人才培养质量,并培养出优质、顶尖、高端的创新型人才,教育部、科技部发布的《教育部科技部关于加强高等学校科技成果转移转化工作的若干意见》中明确提出:高校要及时将科研成果转化为教育教学、学科专业发展资源。因此,处理好教学和科研之间的关系,不仅是政策的驱使,更是提升高校教学质量、增强我国高等教育影响力和活力的重要途径^[2]^。教学与科研是高校教育功能的两大重要组成部分,以培养人才为目标的教学是传授知识的一种最基本形式,科研则是培养创新能力的重要途径。当今世界,经济竞争归根结底是技术竞争,而技术竞争的实质是人才资源的竞争,因此,高校应借教学与科研双重之力,承担起培养有创新能力人才的重任。科研对教学的带动作用以及对学生创新能力的促进作用虽已有较多研究^[3⇓-5]^,但目前仍未得到普遍重视,主要原因是科研与教学之间未能有良好的融合机制^[6]^。将科研成果转化为教学资源是利用科研促进教学的有效途径^[7,8]^。

教学资源,广义上是指在教学过程中可利用的一切要素;狭义上是指教学材料、教学环境及教学后援系统。其中,教材就是最常见的教学材料,是教学过程中物化的资源,具有直观、使用简便的优势,但同时也存在一定的局限性,如知识滞后、篇幅和形式有限、不利于学生个性和创造性发展等,从而影响教学效果和教学质量。与此同时,我国高等教育的发展方向是以提高教学质量为核心的内涵发展^[9]^,因此任何影响教学质量的因素都将成为我国高等教育发展的绊脚石。提高教学质量,首先要从提升教学效果着手,而教学效果的提升与教学资源的丰富程度和合理利用程度有重要关系。

在科技发展日新月异的今天,社会需要的是同时具有丰富知识和创新能力的人才,基于基本教学资源的灌输式教学方法已无法满足这一人才培养的需求。在这种形势下,将先进的科研成果转化为教学资源,通过适当的形式融入教学过程,有助于锻炼学生提出科学问题并解决问题的能力^[10]^。先进的教学资源能够为提升教学效果创造基本条件,不仅能够启发学生通过发现规律和进行科学总结获得解决问题的方法,而且能够提升学生的创新想象力、判断力和思维能力,从而为社会培养知识储备丰富、创新能力突出的专业人才。为了适应经济、科技、文化和社会发展的需要,全面提高本科教学质量和人才培养质量,本着立德树人的教育宗旨和培养复合型高素质专门人才目标,本文以药物分析学课程中色谱方法的教学为例,阐述了如何通过课程设计引入科学发展前沿并渗透科学问题,从而阐明将科研成果转化为教学资源对达成教学目标的促进作用。

## 1 理论部分

### 1.1 药物分析学课程的特点

药物分析学课程是将分析化学、有机化学、药物化学、药剂学等课程通过形成新的综合的知识节点而联系起来的三维知识网,所涉及的知识体系庞大,知识结构灵活^[11]^。尽管学生先学习了必要的基础知识,但是历届学生均反映这门课程知识点繁杂,不容易记牢,尤其是色谱方法在这门课程中涉及面广,药物的鉴别、检查、含量测定等,无一不需要色谱法,要准确掌握其内容难度很大,导致学生期末考试卷面成绩不高、及格率低。我们认为导致上述结果的原因主要有以下几点:色谱法虽然是一种实用性方法,但是由于本科生接触机会少,理解不深入,因此难以将理论知识融会贯通;灌输式教学方式无法提起学生的学习兴趣;师生之间缺乏提出和解决科学问题的探讨过程;学生将零散知识点结成网络的思维导向不正确或能力不足。药物分析学课程的重点内容大都是基于现代化的分析方法和技术,尤其是色谱技术,其更新速度快,理论性与实践性并重,如何设置这张知识网的节点将极大地影响药物分析学课程的可认知程度。

#### 1.1.1 知识体系庞大

药物分析学课程是高等院校药学类专业的必修课程,围绕药物质量控制进行教学,主要研究化学结构明确的合成药物或天然药物及其制剂、中药制剂和生物制品的质量控制方法,旨在通过理论教学与实践操作训练,培养学生具备药品质量全面控制的观念,使学生掌握常用药物的鉴别、杂质检查与含量测定的原理与方法,并具备创新研究和解决药品质量问题的思维和能力。但本课程知识点繁杂,重点和难点多,多种色谱法贯穿始终,导致该课程学习难度较大、不易掌握,这成为影响教学效果和教学质量的关键因素。

#### 1.1.2 知识更新速度快

当今社会正处于“知识大爆炸”的时代,对于药物分析学课程来说更是如此。首先,随着对药品质量要求的提高,药品标准更新较快,如英国药典和美国药典每年更新一次,而且由于标准的提高、方法的改变或新收录的品种等原因,其间还有增补本发行;《中国药典》每5年更新一次,每隔1或2年出版增补本。其次,由于新药的研发与生产不断出现新的质量问题,因此需要研究新的质量控制项目和方法;再次,药学领域中对于药品质量的研究从未停止,色谱-质谱联用技术、毛细管电泳-质谱联用技术、色谱-核磁共振波谱技术、指纹图谱技术等新的分析技术和分析仪器也是日新月异^[12]^。

#### 1.1.3 理论性与实践性并重

药物分析学课程中有关药物的结构和性质、各种分析方法的原理,以及分析方法的选择依据和结果分析等内容是基于大量相关学科的基础知识设置的,具有较强的理论性;与此同时,该门课程培养的是将来能够胜任药品研究、生产、供应和临床使用过程中的分析检验工作,并综合运用所学知识和现代化技术手段、初步制订药品质量标准以及建立新分析方法的人才,因此该门课程同时也具有很强的实践性。

为了提高学生的学习兴趣,帮助学生正确把握和理解该课程的特点和规律,提升教学效果,提高教学质量,并最终达到培养优秀药学类人才的目标,我们将药学学科的前沿进展及课程组教师的先进科研成果转化为教学资源,进行课程设计,融入教学过程,将创新的理念和科研的思路通过渗透的方式传递给学生,不仅有利于学生理解并掌握知识,而且能够引导学生关注药学发展动态,学会以科学的思维方式进行研究。

### 1.2 课程设计

课程设计是以学生为主体,针对如何使学生理解并掌握课程内容的问题,对教学资源进行组织、规划,并形成具有一定评价机制的教学计划和教学大纲的系统化过程。随着我国医药产业的发展,对药学类人才的需求不断增长,同时也对他们提出了更高的要求,课程设计的取向、课程的根本目的及主要任务直接关系到教学效果、教学质量以及培养人才的质量。药物分析学课程作为药学类专业核心课程,其设计取向为技术学取向,但同时由于药物的特殊性,课程设计还应兼顾学术理性主义取向和人本主义取向;课程设计的模式为目标模式,即根本目的是培养优秀的药学类专业人才;课程的主要任务是培养学生药品质量全面控制的观念,使学生掌握常用药物的鉴别、检查与含量测定的原理与方法,并具备创新研究和解决药品质量问题的思维和能力。为了更加清晰,我们用图1展示课程设计场景。

**图1:**
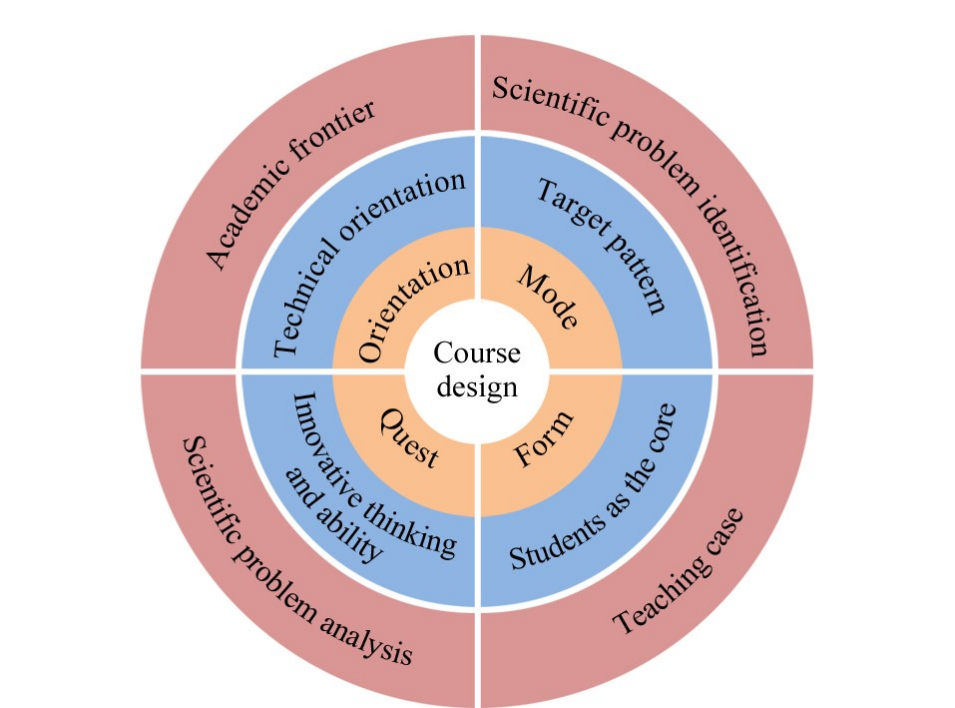
课程设计场景图

药物分析学课程结构主要包括学科发展前沿探究、基本理论知识传授、分组讨论科学问题、实际应用案例分析4个部分。现分别将这4部分的具体设计内容阐述如下。

#### 1.2.1 学科发展前沿的引入

将国际上药物分析学科的发展现状引入课堂,结合教师研究方向,针对热点研究问题进行分析,让学生直观地了解最新研究动态,明确本学科的发展方向以及研究热点的意义和目标。如介绍欧洲及其他发达国家新颁布的药典标准,并与我国药典标准对比,找出差距,明确研究方向;在课堂中引入液相色谱-质谱联用技术的发展现状,尽管目前《中国药典》(2020年版)中使用这一技术的标准有限^[13]^,但是教师应让学生了解目前这种联用技术已经成为前沿研究中最高效的技术之一;同时,为学生介绍学校开放的数据库以及如何查阅并下载相关文献资料,让学生养成自主了解本领域发展前沿的学习习惯;此外,我们还将参加大型学术会议时学者们交流的热点研究内容介绍给学生,这些内容是在他处无法获得的珍贵教学资源,同时通过照片展示,拉近学生与知识的距离,从而更容易产生兴趣并接受,学习科学的思维方式和科学的研究方法。

#### 1.2.2 科学问题的渗透

是否是优秀人才的评判标准之一就是要看其发现问题和解决问题的能力,因此高等教育不仅要使学生掌握知识,更重要的是培养学生发现问题和解决问题的能力,才能培养出社会需要的优秀人才,而简单的知识灌输式授课方式并不利于这种能力的培养。我们将科学问题适时地渗透于基础理论讲授中,不仅有利于加深学生对基础理论知识的理解,而且能够通过科学问题的引导激发学生在基础理论层面之上主动提出问题的积极性,同时还能锻炼学生分析和解决科学问题的能力。例如,结合我们在科研过程中对色谱法的研究,在讲授含量测定等定量分析方法时,提出了“《中国药典》中的含量分析方法通常采用色谱法,而毛细管电泳法也可以进行定量分析,为什么《中国药典》较少采用毛细管电泳法来进行定量分析?”。这一科学问题的提出蕴含着色谱法和毛细管电泳法各自的特点,以此引导学生以分析方法的原理为源头发现问题,并以问题为导向,基于所学知识分析产生这一现象的原因以及解决问题的方法。

#### 1.2.3 科学问题的分析与解决

问题驱动教学法(problem-based learning, PBL)是以学生为主体,学生通过分组形成不同的团队,接受教师分派的不同任务,最终经教师指导、团队协作完成项目,并全程进行评估^[14,15]^。任务中所需要解决的问题,是驱动性问题,即具有可行性、价值性、真实性、意义性、道德性等特点。这种教学方式不仅能够帮助学生将标准化的学科知识学以致用,而且能够引导学生以科学的思维方式发现问题,培养学生解决问题的能力和团队协作能力。此外,问题驱动教学方式非常适合当今社会对人才的培养需求,这种方式有利于加深学生对于知识共享的理解,使其能够更好地融入团队、适应工作环境。例如,我们在药物分析教学过程中设计了10组与色谱法相关的项目,这些项目所涉及的问题均为我们在实际科研过程中所遇到的问题。学生要解决这些问题,不仅需要一定量的多学科交叉理论基础,而且需要查阅文献资料,还需要咨询一些相关专业的教师。课程评价中,学生对问题驱动教学方式普遍给予了极高的评价,认为自己不仅收获了理论知识之外的实践经验,而且锻炼了创新思维方式,提高了自己解决问题的能力。

#### 1.2.4 教学案例

药物分析学课程是河北大学药学类专业培养方案的一门核心课程,设在第7学期,51学时,3学分。近两学年,我们采用了曾苏教授主编的《药物分析学》教材(高等教育出版社,第3版,2022年1月)。尽管学生已在大学第二学年学习了仪器分析课程,但是由于本科生动手操作少,仪器分析实验课程也只是验证性试验,学生只是学到了色谱的理论知识,利用色谱法解决实际问题的能力没有得到充分锻炼,因此很难将其灵活应用于药物分析。此外,虽然也同时开设了药物分析实验课程,但是兼顾知识面,有关色谱法的实验也仅占据极小的一部分。为了锻炼学生利用色谱法解决实际问题的能力,我们结合自己的研究方向,在近三学年药物分析学课程色谱方法的教学中,将新型药物色谱分离介质用于复杂中药体系分析及体内药物分析的研究成果转化为教学资源融入教学过程中,对药物分析的课程设计进行了全面升级:通过在基础知识讲授过程中引入药物分析学科发展前沿,尤其是用于药物分析的新技术和新方法的开发,如色谱-质谱联用技术、超高效液相色谱技术以及激光诱导荧光等新型检测技术,让学生了解学科发展现状以及当前热点研究方向,激发学生探索创新的兴趣;在教学过程中渗透相关科学问题,如在讲授体内药物分析时,引入生物样品前处理的科学问题、各种前处理技术的特点以及如何根据样品性质开发先进的前处理方法,从而在已有的基础理论知识上增加其“灵活”和“可开发”性,引导学生积极思考,并利于学生养成提出问题的学习习惯;在一定知识范畴内,设定符合学生研究能力的项目,通过问题驱动教学模式让学生主导开展问题讨论和推进,学生要运用自己储备的知识网络,兼顾项目的整体与局部,分析问题并解决问题,由此形成科学的思维方式,积累发现问题、解决问题的经验,提升实践能力^[16]^。如我们在授课前事先拟好了10个自主设计实验的项目(见表1),借助超星泛雅在线教学平台,将学生分为10组,每组随机抽取一个项目,项目设计内容为采用最新技术或方法对给定的几类药物或样品进行定性和定量分析方案设计,说明设计原理、可行性、可能遇到的问题与解决方案以及项目组成员的讨论纪要等,并将最终方案形成报告在线提交,期限为4周。

**表1:** 10组PBL项目

Nos.	Project (Title)	Score (×coefficient)
1-7	Based on the structure and properties of select compounds, two methods (at least one of which is a chromatographic method) for identification and one chromatographic method for content determination should be designed by referring to the literature and combining previous knowledge. The structural formulas are as follows: 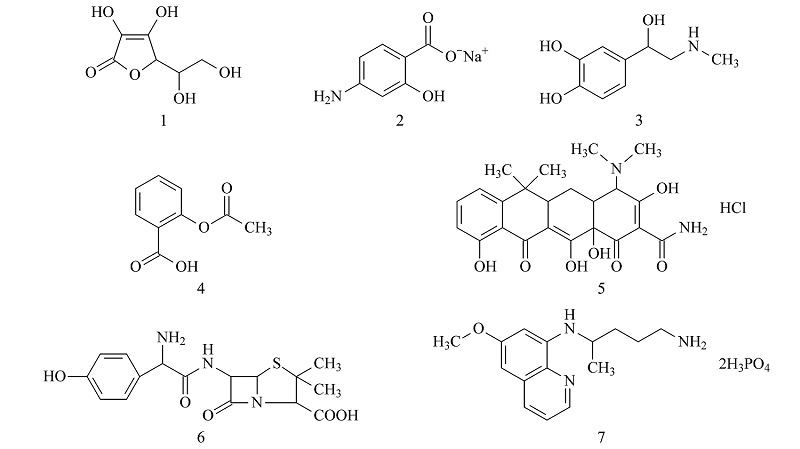	100 (×0.9)
8	An HPLC fingerprint analysis scheme should be designed to analyze the active components of a Chinese medicine without obvious UV absorption.	100 (×1.0)
9	An HPLC scheme should be designed for the qualitative and quantitative analyses of five anthraquinones in Rheum palmatum L. (rhein, emodin, chrysophanol, physcion, and aloe-emodin).	100 (×1.0)
10	A chromatography-based method, together with a sample-pretreatment method, should be designed to enable the study of the metabolism of phenobarbital in mouse blood.	100 (×1.0)

在此期间,我们也不同程度地参与了每一组的讨论,发现学生能够通过查阅文献解决部分问题,并能利用所学知识经过反复论证形成最终方案。评分由教师评分(权重50%)和项目组互评(权重50%)组成。PBL模式项目的开展,不仅增加了学生与教师的互动,增强了团队的凝聚力,让学生意识到团队协作的意义,有利于日后快速融入工作团队,更让学生领会了如何充分利用前人的最新科研成果来解决科学问题。

本课程组近10年一直使用题库模式进行期末考试,虽然期间有更新,但难度系数均控制为0.55~0.65,并且有统一的评分标准,因此学生的成绩可以从一定程度上反映教学效果和教学质量。在实施“科研成果转化教学资源”近3个学年,学生成绩有了很大程度的提高:最高分从实施之前的77分提高到92分,期末考试成绩不及格率从25%降至2%。此外,学生在评教系统中对药物分析学课程的教学方式和效果给出了高度评价,97%以上的学生认为学到了书本以外的知识,开拓了思维,提升了自己解决问题的能力。

## 2 结果与讨论

### 2.1 课程考核

对教学效果进行评估,是对课程设计及教学方式的总结与检查,对进一步完善课程设计和丰富教学方式有一定的促进作用。传统的考核方式仅能考查学生对各类药物的性质、各种分析方法的原理以及《中国药典》中采用的具体的分析方法等的掌握程度,这些知识体现的主要是学生的记忆力,而对于学生提出问题、分析问题、针对新药建立新的分析方法的能力则展现不足,既无法真实地体现将科研成果转化为教学资源的教学效果,也不符合对培养优秀人才的考核指标。针对上述问题,结合河北大学对于课程考核的规定及教学大纲要求,我们丰富了平时成绩(总成绩=期末成绩×70%+平时成绩×30%)的项目,除“作业”“课堂测验”“考勤”外,还增加了“课堂互动”和“PBL分组讨论”两项内容。这两项在平时成绩中的占比为50%,并且贯穿整个教学过程,因此也更能反映学生掌握并灵活运用药物分析技术的真实水平。

### 2.2 学生反馈

学生反馈是学生对教师业务水平、教学方法、教学态度的评价,同时也是学生对自己学习效果满意程度的表达,因此能够比较直接地反映教学效果。在教学过程中将科研成果转化为教学资源后,我们所授课班级学生匿名反馈结果统计数据表明:学生针对教学内容中“老师注重吸收学科新知识、新成果,及时介绍前沿动态,拓宽了我的知识圈。”单项评分为9.93分(满分10分);针对教学效果中“通过学习,我的学习兴趣和学习热情得到激发。”单项评分为9.90分(满分10分)。这表明绝大多数学生对于充实了新的教学资源的新教学方法是非常认同和满意的。

### 2.3 自我评价

自我评价是教师本人对教学目标达成程度和学生学习效果的评价,是教师自我认识、自我反思、自我提高的必要过程。通过教师自我评价,能够从根本上发现教学资源与教学方式的优势与不足,从而在以后的教学中扬长补短,达到提高教育教学质量的目的。我们将科研成果转化为教学资源并采用多种形式进行教学,收到了良好的教学效果,在一定程度上提高了学生提出问题、解决问题的能力,激发了学生的学习积极性和作为教学主体的课堂主动性,学生期末成绩有明显提升且反馈结果良好。

## 3 结语

为了提升教学效果,提高教学质量,达成药物分析学课程教学目标,最终实现优秀药学类人才的培养,基于药物分析学课程的特点,我们将药学学科的色谱领域前沿进展及科研成果转化为教学资源,进行课程设计,融入教学过程,并采用多种适当的教学手段实施教学,不仅能够充分利用资源,弥补教材的不足,而且有利于丰富教学形式,激发学生的求知欲和提高学生的学习积极性。

尽管如此,要将“科研成果转化为教学资源”在教学中传承并发展下去,还需要克服一些难题。首先是科研成果与教学资源的相融性问题,并不是每一位任课教师的研究方向都与其所承担的课程相符,这会导致其科研成果无法恰当地转变为该课程的教学资源。其次,对于一些百家争鸣的学术问题,教师的科研成果存在一定的主观性,如果教师没有正确认识或没有合理运用,不利于学生判断力的提升,而且容易导致学生接受偏颇的学术知识。最后,学生对由科研成果所转化的教学资源接受能力不同,存在个体差异,教师授课时一概而论则容易事倍功半。因此,教师需要合理运用并正确引导学生、因材施教,才能使“科研成果转化为教学资源”的方式在教学中发挥最大优势,最终起到提升教学效果、培养药学优秀人才的目的。
